# Determinants of PrEP Delivery Platform Choice in Adolescents and Young People: The Fast-PrEP Project in Cape Town, South Africa

**DOI:** 10.3390/ijerph23081045

**Published:** 2026-08-12

**Authors:** Elzette Rousseau, Connor Bondarchuk, Keitumetse Lebelo, Luyanda Matsebula, Fiona Bennin, Pippa Macdonald, Carey Pike, Onesimo Vanto, Ntombovuyo Mathole, Lauren Fynn, Dvora Joseph Davey, Chelsea Coakley, Melissa Wallace, Linda-Gail Bekker

**Affiliations:** 1 Desmond Tutu HIV Centre, Institute of Infectious Diseases and Molecular Medicine, University of Cape Town, Cape Town 7925, South Africa; 2 Harvard Medical School, Boston, MA 02115, USA; 3 Division of Epidemiology and Biostatistics, School of Public Health, University of Cape Town, Cape Town 7701, South Africa; 4 Department of Infectious Diseases, Geffen School of Medicine, University of California, Los Angeles, CA 90095, USA; 5 Centre for Social Science Research, University of Cape Town, Cape Town 7701, South Africa

**Keywords:** mobile clinics, pre-exposure prophylaxis (PrEP), adolescents and young people (AYP), differentiated service delivery

## Abstract

**Highlights:**

**Public health relevance—How does this work relate to a public health issue?**

- Effective HIV prevention in South Africa requires implementing pre-exposure prophylaxis (PrEP) service delivery options responsive to adolescents and young people (AYP) with diverse characteristics and needs.
- De-medicalised, differentiated, community-based platforms like mobile clinics for PrEP delivery might reach more AYP who may benefit from PrEP.

**Public health significance—Why is this work of significance to public health?**

- Findings support AYP’s strong preference for community-based HIV prevention services (mobile clinics) and emphasise the need for accessibility and service location autonomy.
- AYP’s broader SRH (sexual and reproductive health services) needs influence their choice of PrEP service delivery platform, with public health facilities better positioned to serve more complex healthcare needs.

**Public health implications—What are the key implications or messages for practitioners, policy makers and/or researchers in public health?**

- HIV prevention programmes should offer both community-based and public health facility PrEP delivery options to maximise accessibility and meet the diverse healthcare needs of AYP.
- AYP-responsive PrEP services should incorporate mental health support, alcohol use screening and broader SRH to improve HIV prevention engagement and outcomes.

**Abstract:**

Background: Effective HIV prevention in South Africa requires implementing pre-exposure prophylaxis (PrEP) service delivery options responsive to adolescents and young people (AYP) with diverse characteristics and needs. We aimed to identify characteristics associated with AYP’s choice of PrEP initiation setting. Methods: Fast-PrEP is an ongoing phase 4 HIV prevention programme in Cape Town, South Africa. Oral PrEP (TDF/FTC) is offered to AYP (15–29 years) within sexual and reproductive health services from four community-based mobile clinics and 12 public primary healthcare facilities. Descriptive statistics and logistic regression models evaluated sociodemographic, behavioural, and clinical factors associated with PrEP initiation from mobile clinics and public health facilities in 13,807 AYP between Aug’22 and Dec’24. Results: Most AYP (70.7%; *n* = 9760) initiated PrEP from mobile clinics. AYP with more complex healthcare needs, such as pregnancy, parenthood, known sero-different relationships, or currently having STI symptoms, were significantly more likely to use the public health facilities. Both male and female AYP initiating PrEP at mobile clinics had four times the odds of moderate-to-severe depression. Previous PrEP experience was not associated with the choice of PrEP service delivery platform. Conclusions: Our results indicate that offering community-based PrEP alongside public sector facilities increases the reach of a diverse group of AYP who may benefit from PrEP.

## 1. Introduction

More than 40 million people are living with HIV, and global targets for reducing new HIV infections have been consistently unmet due to inequalities in HIV programme accessibility [1,2]. Pre-exposure prophylaxis (PrEP) has been a transformative advancement in HIV prevention. However, by 2024, only 69% of 194 countries (*n* = 133; with missing country data for *n* = 47) had approved at least one PrEP technology (primarily oral PrEP), and <50% (*n* = 93; with missing country data for *n* = 22) had provided access to all population groups [3]. Sub-optimal PrEP uptake has also been observed in East and Southern African (ESA) countries, where PrEP has been approved for several years [4,5]. While South Africa has scaled up oral PrEP to most primary healthcare public health facilities, with >1.6 million people initiating PrEP since 2015, approximately 3500 people still acquire HIV every week [6,7]. Factors limiting PrEP initiation and optimal use in ESA include healthcare system barriers such as provider attitudes and insufficient training to discuss and prescribe PrEP; structural healthcare access challenges (distance to facilities, waiting times, and inconvenient clinic hours); limited awareness and stigma in communities that raise concerns about unintentional disclosure or intimate partner violence (IPV); and low risk perception or social support for PrEP use, especially in young people [8,9,10,11,12,13,14,15,16].

In 2022, the World Health Organisation (WHO) published a technical report encouraging differentiated service delivery approaches that support PrEP uptake and continued use by making services more accessible and acceptable to all populations who may benefit from PrEP [17]. The WHO highlights that “the way services are organised and provided can either limit or enable access”, and AYP, especially, have indicated a preference for services that fit into their lifestyles and routines [18]. Recent delivery models supporting increased PrEP accessibility have included the integration of PrEP into family planning [19,20,21] or antenatal care services [22,23,24], community-based mobile clinics [25,26], private pharmacies [27], the inclusion of HIV self-testing [28], and peer-delivery approaches [29,30,31,32,33].

While various differentiated delivery approaches have been adopted in recent years, there is limited evidence on (1) young people’s preferences when offered a choice of PrEP delivery platforms and (2) the effectiveness of different PrEP delivery platforms in reaching young people who may benefit from PrEP [17]. Fast-PrEP is an ongoing programme designed to scale up and evaluate oral PrEP (TDF/FTC) delivery to AYP from community-based mobile clinics, government public health facilities, and schools in Cape Town, South Africa [34]. In this manuscript, we evaluated the association of AYP’s sociodemographic, sexual behaviour, clinical and PrEP-related characteristics with their choice of PrEP initiation at community-based mobile clinics versus public health facilities. This paper focused on AYP’s delivery site engagement at the time of PrEP initiation only.

## 2. Materials and Methods

### 2.1. Research Setting and Study Participants

The Fast-PrEP programme in Cape Town, South Africa, provided integrated sexual and reproductive health (SRH) services, including oral PrEP, to young people focusing on adolescent girls and young women (AGYW; 15–29 years old), pregnant and breastfeeding people (PBFW), men who have sex with men (MSM) and intimate male partners (IMP; 15 years and older). Participants were recruited from self-presenting AYP during individual SRH consultations at Fast-PrEP mobile clinics and participating public health facilities in the Klipfontein Mitchells Plain health sub-district. Eligibility criteria included an HIV-negative test result, self-identification as a candidate for PrEP, interest in initiating PrEP for HIV prevention on that day, meeting South Africa’s national PrEP eligibility criteria, and the ability and willingness to provide electronic informed consent for data collection. People with existing HIV infection or those newly testing HIV-positive were ineligible for enrolment and were referred for treatment and care at nearby public health facilities. Study procedures have been described in detail previously [34]. Before the study commenced, ethical clearance was obtained from the University of Cape Town (HREC 713/2021), the City of Cape Town Department of Health (#9522), and the Department of Basic Education (#20220609-2975). Prior to data collection, participants were informed about the focus and procedures of the research, and electronic informed consent was obtained in English or isiXhosa (predominant local language). Parental proxy consent was waived in consultation with the ethical review board and community advisors for participants aged 15–17 years old (18 years is considered the age of adulthood in South Africa).

### 2.2. PrEP Delivery Platforms

**Figure 1:**
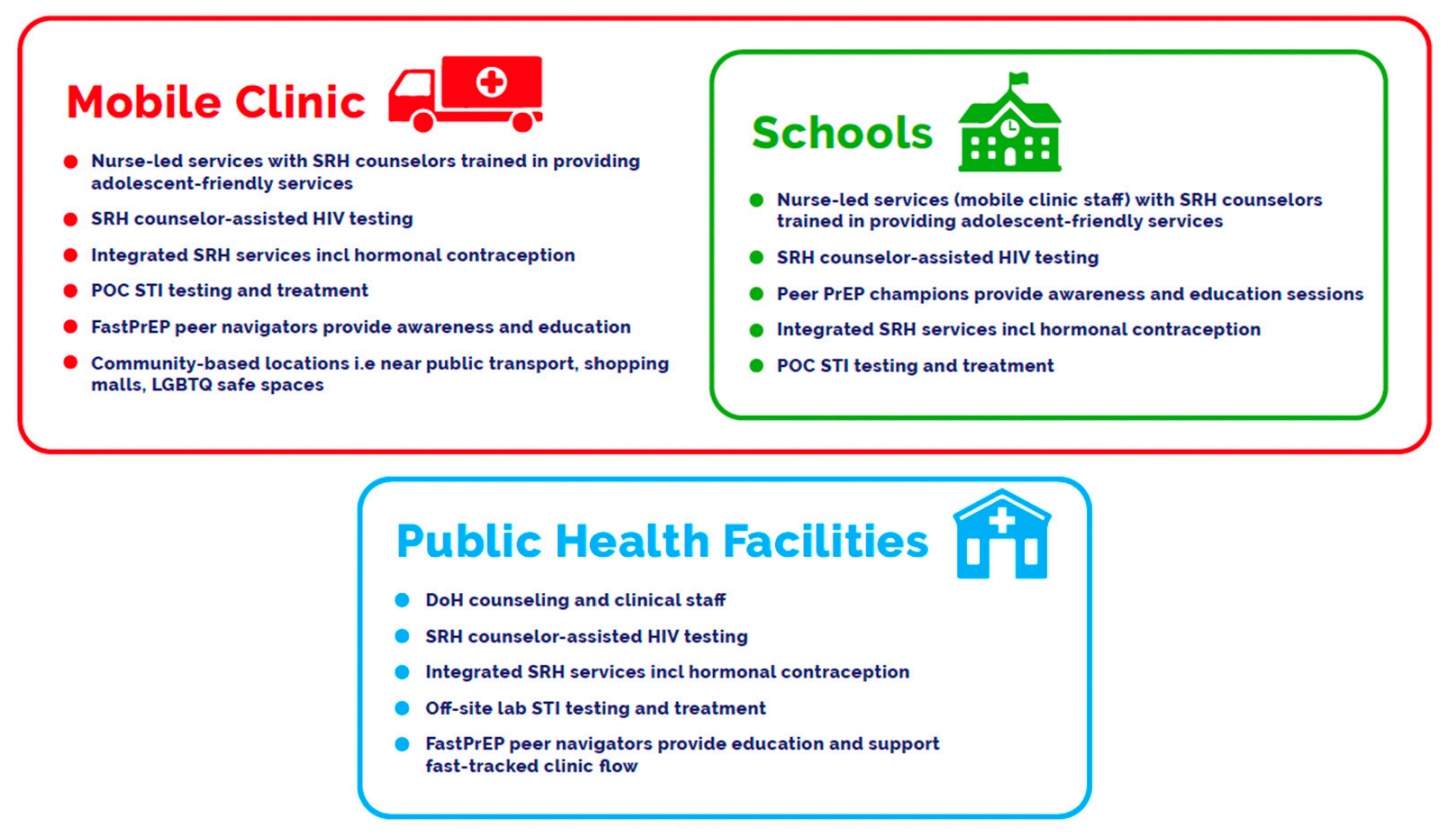
Fast-PrEP project delivery platforms. Young people initiate PrEP within integrated SRH services from nurse-led community mobile clinics (which also provided services at select schools) and public health facilities with peer navigator support.

Young people could initiate PrEP from four community-based mobile clinics or 12 fixed-site government public health facilities in the Klipfontein Mitchells Plain health sub-district (Figure 1).

Mobile clinics (*n* = 4) provided oral PrEP as part of a nurse-led service staffed with SRH counsellors, peer navigators, and a driver. Peer navigators (trained lay young people aged 18–29 years) provided awareness and education on SRH, including PrEP, outside the mobile clinics. The nurses had medical clinician support available by telephone if needed. All staff working in the mobile clinics were trained in providing adolescent-friendly and gender-inclusive services. The mobile clinics provided point-of-care (POC) HIV and pregnancy testing; STI testing and treatment; and offered a range of hormonal contraception options (oral, injectable and implant). Services were provided in community settings, including public transport spaces, shopping malls, key population safe spaces and schools. At schools (*n* = 16), mobile clinic staff and peer PrEP champions first provided awareness and education sessions to students, followed by service provision on school premises. The Fast-PrEP mobile clinics’ schedule was posted weekly on social media.

Fixed-site public health facilities (*n* = 12) were each supported by two Fast-PrEP peer navigators (*n* = 24 total; aged 18–29 years) providing SRH and PrEP education [35], while the counselling and clinical staff were existing Department of Health (DoH) facility staff. Public health facilities offered a range of SRH, HIV, and antenatal care (ANC) services (full process described previously) [34] (Figure 1).

During the enrollment visit, AYP were informed of all the alternative delivery sites for their PrEP refills. A biometric tracking system was utilised to track participants’ usage of the different delivery sites from initiation and through all follow-up visits (described previously) [34].

### 2.3. Data Collection and Study Variables

The primary outcome was the PrEP initiation site, categorised as a mobile clinic (including school-based mobile services) or a fixed-site government public health facility. PrEP initiation was defined as receipt of a 28-day supply of oral PrEP tablets. Data were collected at the enrollment visit and managed using REDCap electronic data tools.

Candidate explanatory variables included sociodemographic, behavioural, clinical and PrEP-related characteristics. Demographic characteristics included age group (15–19, 20–24, 25–29, >29), sex, relationship status (married/single), and parental status (number of children). Behavioural characteristics included participants’ self-reported knowledge of a partner living with HIV (yes/no/do not know), engagement in casual sex in past month (yes/no), multiple concurrent sexual partners in the past month (yes/no), having a partner ≥ 10 years older or younger (yes/no/do not know), and whether participants believed their sexual partners had other partners (yes/no/do not know). Clinical characteristics included depressive symptoms, hazardous alcohol use, current STI symptoms (yes/no), having an STI or a partner with an STI in the past month yes/no), and pregnancy status for females. Depression and anxiety were measured with the PHQ-4 (the four-item Patient Health Questionnaire) [36,37] and dichotomised as showing no/mild (score of 0–5) or moderate/severe (score ≥ 6) signs of mental health concerns. Hazardous alcohol consumption was assessed using the AUDIT-C tool (Alcohol Use Disorder Identification Test–Concise) [38,39,40] and defined by a score of ≥3 for women or ≥4 for men. PrEP-related characteristics included prior PrEP use (yes/no), disclosure of intended PrEP use, and anticipated partner reaction to PrEP (positive/negative/unsure).

### 2.4. Statistical Analysis

Descriptive statistics were generated for three sets of baseline characteristics, including (1) sociodemographic and behavioural characteristics (including characteristics related to relationship dynamics); (2) clinical (including mental health) characteristics; and (3) PrEP-related characteristics. We hypothesised (based on previous literature on PrEP uptake) that factors related to these characteristics may influence the choice of PrEP initiation setting. Categorical variables were summarised using frequencies and percentages and compared between participants initiating PrEP at mobile clinics and fixed-site public health facilities using Pearson’s χ^2^ tests. To reflect sex-specific differences, these comparisons were performed separately among male and female participants. Continuous variables were summarised using means (standard deviations) and compared between service delivery platforms within each sex using Student’s *t*-tests.

Univariable logistic regression models were initially fitted to estimate the association between each characteristic and choice of PrEP initiation site. Variables were selected a priori based on clinical relevance and previous literature and entered simultaneously within each conceptual domain. Subsequently, multivariate logistic regression models were used to explore the relationship between each set of baseline characteristics (sociodemographic and behavioural, PrEP-related characteristics, and clinical characteristics) and PrEP initiation at a mobile clinic. Odds ratios (OR) and adjusted odds ratios (aOR) are presented with 95% confidence intervals (95% CI). Model fit was assessed using the Hosmer-Lemeshow goodness-of-fit test. Model discrimination was evaluated using the area under the receiver operating characteristic curve (AUC). Multicollinearity was assessed using variance inflation factors (VIF), with no evidence of problematic collinearity (mean VIF = 1.66 all VIFs < 3). To assess the potential impact of missing data, a sensitivity analysis using multiple imputations by chained equations was performed. The results were consistent with those of the complete-case analysis.

IBM SPSS Statistics Version 30.0.0.0 was used for all analyses. Missing data were handled using complete-case analysis for regression models.

## 3. Results

### 3.1. Demographic, Behavioural and Clinical Characteristics of Cohort Initiating PrEP

A total of 13,807 people initiated oral PrEP between August 2022 and December 2024. The cohort had a median age of 23 (21 years for AGYW and 28 years for IMP), with the majority (58.6%; *n* = 8092) between the ages of 15–24. Most were AGYW (58.5%; *n* = 8082) or IMP (36.3%; *n* = 5016); followed by men who have sex with men (MSM; *n* = 317, 2.3%) and pregnant people (*n* = 392, 2.8%). The cohort was mostly unmarried (94.4%), with about half having ≥1 child (46.2%; *n* = 5101). The four community-based mobile clinics were the PrEP initiation platform for 70.7% (*n* = 9760; with *n* = 259 (2.7%) initiations on public school grounds) compared to 29.3% (*n* = 4047) of people initiating across the 12 fixed-site public health facilities (Table 1).

Table 2 presents the behavioural, clinical and PrEP-related characteristics of the PrEP cohort among participants with available data for each measure. Among those with available data, 3.9% (*n* = 506) were in sero-different relationships (as high as 16.1% for men accessing public health facilities) and 5.3% (*n* = 689) were in age-disparate relationships. Most participants (59.7%; *n* = 7830) were unsure whether their primary sexual partner had other partners, and half (51.0%; *n* = 6691) did not know their partner’s serostatus. While 13.3% (*n* = 1039) of females were in multiple concurrent relationships, 36.7% (*n* = 1902) of all men (with as high as 43.6% of those attending public facilities; *p* < 0.001) had multiple partners in the past month. During the month before PrEP initiation, 30.4% (*n* = 1084) of men attending mobile clinics and 42.8% (*n* = 488) of men at public health facilities (*p* < 0.001) engaged in casual sex compared to 16.5% (*n* = 787) and 13.5% (*n* = 305), respectively, in the female cohort.

Moderate-to-severe depressive symptoms were reported by 11.9% (*n* = 872) of participants attending mobile clinics and 2.6% (*n* = 66) of those attending public facilities. Across PrEP delivery sites, more than half (57%; *n* = 4602) of men and women displayed hazardous alcohol use levels. Among participants with available STI-related data, 8.5% (*n* = 1110; and as high as 22.5% of men at public health facilities; *p* < 0.001) reported that they or a sex partner had an STI in the past month, and 10.4% (*n* = 1078) displayed symptoms of an STI at the PrEP initiation visit (28.3% of men at public health facilities; *p* < 0.001).

The majority of the cohort was PrEP-naïve, with only 17.3% (*n* = 2336) having used PrEP prior to enrolment into Fast-PrEP. However, 41.4% (*n* = 3357) of participants for whom data were available indicated that they had disclosed their intention to start using PrEP to someone. While 54.6% predicted that their sex partner would have a positive reaction to their PrEP use, 38.2% were unsure what their partner’s reaction would be, and 7.2% expected a negative reaction (Table 2).

### 3.2. Sociodemographic, Behavioural and Clinical Characteristics Associated with PrEP Service Delivery Platform Choice

When comparing people who initiated PrEP from a community-based mobile clinic with those who initiated PrEP at government public health facilities (Table 3), those at the mobile clinic were significantly more likely to be older than 15–19 years, and less likely to have a child (aOR 0.560, 95% CI: 0.55 to 0.66, *p* < 0.001) or be single (unmarried) (aOR 0.746, 95% CI: 0.60 to 0.92, *p* = 0.007). Females were less likely to initiate PrEP at a mobile clinic (aOR: 0.696, 95% CI: 0.62 to 0.78, *p* < 0.001). People in a known HIV sero-different relationship (aOR 0.276, 95% CI: 0.22 to 0.34, *p* < 0.001) were less likely to initiate PrEP at the mobile clinic, while people who were unsure of their partner’s HIV status were more likely to use this platform (aOR 1.417, 95% CI: 1.30 to 1.55, *p* < 0.001). Neither participants nor their sex partners having other sex partners or having casual sex in the past month was significantly associated with PrEP service delivery platform engagement.

Participants currently showing symptoms of an STI or whose primary sex partners had an STI in the past month were significantly associated with lower odds of initiating PrEP at a mobile clinic for both male (aOR 0.278, 95% CI: 0.22 to 0.36, *p* < 0.001; aOR 0.585, 95% CI: 0.43 to 0.79, *p* < 0.001) and female participants (aOR 0.732, 95% CI: 0.55 to 0.97, *p* = 0.030; aOR 0.634, 95% CI: 0.46 to 0.88, *p* = 0.006).

Both men and women initiating PrEP at the mobile clinic had four times the odds of having symptoms of moderate-to-severe depression (aOR 3.892, 95% CI: 2.44 to 6.22, *p* < 0.001; and aOR 4.102, 95% CI: 2.56 to 6.57, *p* < 0.001, respectively). Pregnant women were significantly less likely to initiate PrEP from a community-based mobile clinic compared to a fixed-site public health clinic (aOR 0.089, 95% CI: 0.05 to 0.15, *p* < 0.001). Hazardous alcohol use was not significantly associated with PrEP initiation location.

People accessing PrEP from the mobile clinic were more likely to be unsure of their partner’s reaction to their PrEP use (aOR 1.863, 95% CI: 1.62 to 2.14, *p* < 0.001) or predict that their partner would have a positive reaction (aOR 4.528, 95% CI: 3.94 to 5.20, *p* < 0.001) compared to a negative partner reaction. Previous PrEP experience was not significantly associated with the PrEP service delivery platform (aOR 1.085, 95% CI: 0.98 to 1.21, *p* = 0.126).

## 4. Discussion

The Fast-PrEP phase 4 programme in South Africa attracted a diverse group of adolescents and young people and corroborates the benefit of a differentiated HIV prevention delivery service to ensure greater coverage of populations who can benefit from PrEP. This has also been shown in other models [17,41,42]. While participants were allowed to self-determine whether PrEP was appropriate for them, most reported multiple factors associated with HIV acquisition, including having multiple concurrent sex partners, casual sex partners, not knowing sex partners’ HIV status, and/or hazardous alcohol use.

AYP in our study showed a strong preference for community-based HIV prevention services (70.7% of the Fast-PrEP cohort initiated at mobile clinics), similar to studies conducted across southern Africa, emphasising the need for accessibility and service location autonomy [21,25,33,43]. People accessing community-based services, including mobile clinics, expect to receive youth-friendly, non-judgmental, and flexible services [21,25,44]. Our study showed that young people accessing mobile clinics appropriately identified themselves as candidates for PrEP and were more likely to be unsure of their partners’ HIV status. Men (intimate male partners) were more likely to initiate PrEP at mobile clinics compared to public facilities, which confirms the limited qualitative research on this population’s PrEP uptake facilitators [45,46]. In addition, young people accessing PrEP from a mobile clinic were also more likely to predict that their partners would be supportive of their PrEP use (i.e., expecting less stigma for service attendance and PrEP initiation). Interestingly, young people seeking out community-based mobile clinics were four times more likely to have symptoms of moderate-to-severe depression. In South Africa, there is a high burden of depression among young people seeking SRH services [47]. Previous research has shown that help-seeking behaviour among adolescents and young people with depressive symptoms is influenced by perceived service accessibility and ease of access to care providers, short waiting times, and support from peers, and it is notable that they found the community-based, mobile services to be more accessible than public sector clinics in this programme [48,49].

Young people with more complex healthcare needs beyond SRH, such as those who were pregnant or had children, were in known HIV sero-different relationships, had STI symptoms, or recently had an STI in their relationship (themselves or their partner), opted to initiate PrEP in public health facilities. While evidence to support a preference for government public health facilities is limited, some participants in studies from South Africa and Namibia appreciated the professional oversight, infrastructure, integration and access to other services [44,50,51]. Participants’ previous or co-morbid conditions may have meant they had already accessed the public sector service, facilitating re-engagement for prevention services. The candidacy framework [52] (used previously in South African contexts [53,54,55]) recognises that perceived eligibility (candidacy) for an intervention or service is shaped by social and contextual factors. First, a person determines whether they are a good candidate for an intervention (i.e., PrEP), then decides where to go (community or public health facility) based on their past experiences and the service’s signals about who is a good candidate for that delivery platform [52]. A similar pattern emerged in our study. Pregnant people and mothers were more likely to access public health facilities, underscoring the need to prioritise targeted PrEP engagement in antenatal, postnatal and early childhood care services [23,56,57,58,59,60].

Our results indicated that the choice of PrEP service delivery location was influenced by clinical characteristics and perceived health needs rather than prior PrEP use experience. In summary, we found that the majority of AYP initiating oral PrEP from mobile clinics were driven by general SRH needs, while AYP with other healthcare needs beyond SRH initiated PrEP at public health facilities. Of note, our study demonstrated that more than half of participants displayed hazardous alcohol consumption (even though it was not significantly associated with a specific PrEP delivery site). Alcohol use may contribute to HIV acquisition and may undermine PrEP use (including delayed refill visits) [61,62,63,64]. It is important that places where PrEP is offered incorporate screening and brief counselling, and long-acting PrEP formulations to support this key population who typically avoid healthcare systems or struggle to effectively use these interventions [61,65,66].

The strength of our study is the large cohort of young people initiating PrEP in a real-world setting that simulated a free, government service with no study-specific reimbursement offered, allowing identification of factors associated with PrEP service delivery platform choice not previously assessed at scale and outside of a controlled study setting. However, the PrEP delivery setting might have been biased geographically by proximity of services to young people with mobile clinics available near schools, malls and major transport hubs. While the public health facilities in this study provided enhanced adolescent-friendly services through the availability of peer navigators, this is not the reality in other government facilities. Given the large sample size, even relatively modest associations may achieve statistical significance. Therefore, the findings should be interpreted based on the magnitude and precision of the adjusted odds ratios, rather than statistical significance alone. Furthermore, because this was a cross-sectional analysis, the reported associations should not be interpreted as causal relationships. Residual confounding and unmeasured factors may have influenced the observed associations despite adjustment for measured covariates. A further limitation of this study was that several of the behavioural and clinical variables, including PHQ-4 and AUDIT-C measures, had substantial and differential missingness across service delivery platforms (for the first year of study implementation, these scales were only administered to every 4th participant), which may have influenced the magnitude of observed associations. Further, while the study initiated a large cohort of young people, these findings may not be generalisable beyond high-density peri-urban settings in South Africa.

## 5. Conclusions

Most adolescents and young people prefer and use community-based PrEP delivery that is easily accessible from mobile clinics and public schools. Our study also showed the benefit of providing PrEP delivery platform choice and integrated SRH services to reach a greater diversity of populations that may benefit from a variety of prevention needs, including HIV, and demonstrated how young people continuously assess the perceived accessibility and appropriateness of healthcare systems (delivery platforms) for their needs. Sometimes this goes beyond HIV prevention, highlighting the importance of integrated health delivery and ensuring that demand generation for PrEP services highlights the availability of other health interventions as well. Ultimately, service delivery models that include both community-based and public health fixed facility platforms may be needed to provide PrEP (including newer longer-acting methods) at scale to all young people who may benefit from HIV prevention.

## Figures and Tables

**Table 1:** Sociodemographic characteristics of cohort by PrEP initiation site.

Baseline Characteristic	PrEP Initiation at Mobile Clinic (*n* = 9760) *	PrEP Initiation at Fixed-Site Public Health Facilities (*n* = 4047)	Total Initiated on PrEP (*n* = 13,807) *
Median age, IQR	23 (19–28)	22 (19–27)	23 (19–28)
Age cohorts (*n*, %)			
15–19	2448 (25.1%)	1187 (29.3%)	3635 (26.3%)
20–24	3087 (31.6%)	1370 (33.8%)	4457 (32.3%)
25–29	2481 (25.4%)	1004 (24.8%)	3485 (25.2%)
>29	1744 (17.9%)	485 (12.0%)	2229 (16.1%)
Missing	0	1	1
Population (*n*, %)			
AGYW (female, 15–29 years)	5597 (57.3%)	2485 (61.4%)	8082 (58.5%)
IMP (male, ≥15 years)	3862 (39.6%)	1154 (28.5%)	5016 (36.3%)
MSM	211 (2.2%)	106 (2.6%)	317 (2.3%)
Pregnant or Breastfeeding People	90 (0.9%)	302 (7.5%)	392 (2.8%)
Missing	0	0	0
Relationship Status (*n*, %)			
Married	596 (6.1%)	153 (4.3%)	749 (5.6%)
Single with partner (not married)	9122 (93.9%)	3429 (95.7%)	12,551 (94.4%)
Missing	42	465	507
Number of living children (*n*, %)			
None	4286 (55.7%)	1647 (49.3%)	5933 (53.8%)
≥1 child	3404 (44.3%)	1697 (50.7%)	5101 (46.2%)
Missing	2070	703	2773

* Note percentages reflect non-missing observations.

**Table 2:** Behavioural and clinical characteristics of cohort at PrEP initiation, stratified by PrEP initiation site and gender.

Baseline Characteristic	PrEP Initiation at Mobile Clinic (*n*, %) *		PrEP Initiation at Fixed-Site Public Health Facilities (*n*, %) *		*p*-Value **	
Behavioural Characteristics						
	Male	Female	Male	Female	Male	Female
Sex partner(s) living with HIV (*n* = 13,149)						
Yes	188 (4.7%)	70 (1.3%)	187 (16.1%)	61 (2.6%)	<0.001	<0.001
No	1742 (43.1%)	2539 (45.5%)	458 (39.5%)	1184 (50.5%)		
Unsure	2109 (52.2%)	2966 (53.2%)	516 (44.4%)	1100 (46.9%)		
Missing	18	109	88	441		
Partner ≥ 10 years older/younger than yourself (*n* = 13,137)						
Yes	231 (5.7%)	195 (3.5%)	69 (5.9%)	194 (8.3%)	<0.001	<0.001
No	3754 (93.1%)	5310 (95.3%)	1045 (90.0%)	2097 (89.5%)		
Unsure	49 (1.2%)	65 (1.2%)	47 (4.1%)	52 (2.2%)		
Missing	23	114	88	443		
Multiple partners in past month (*n* = 13,047)						
Yes	1404 (34.8%)	737 (13.3%)	498 (43.6%)	302 (13.3%)	<0.001	0.479
No	2596 (64.3%)	4780 (86.0%)	637 (55.7%)	1954 (85.7%)		
Unsure	38 (0.9%)	41 (0.7%)	8 (0.7%)	23 (1.0%)		
Missing	19	126	106	507		
Engaged in casual sex in past month (*n* = 11,767)						
Yes	1084 (30.4%)	787 (16.5%)	488 (42.8%)	305 (13.5%)	<0.001	0.001
No	2478 (69.6%)	3989 (83.5%)	642 (57.2%)	1956 (86.5%)		
Missing	495	908	109	525		
Partner has other sexual partners (*n* = 13,142)						
Yes	254 (6.3%)	393 (7.1%)	76 (1.4%)	207 (8.8%)	0.362	<0.001
No	1401 (34.7%)	1852 (33.2%)	4727 (86.6%)	673 (28.7%)		
Unsure	2382 (59.0%)	3328 (59.7%)	658 (12.0%)	1462 (62.4%)		
Missing	20	111	88	444		
Clinical Characteristics						
Mean PHQ-4 Score, SD (*n* = 9855)	1.43, 2.26	1.69, 2.42	0.93, 1.70	0.84, 1.64	<0.001	<0.001
Depressive Symptoms (PHQ-4) (*n* = 9855)						
None-Mild	2734 (86.5%)	3708 (89.3%)	806 (97.0%)	1642 (97.6%)	<0.001	<0.001
Moderate-Severe	428 (13.5%)	444 (10.7%)	25 (3.0%)	41 (2.4%)		
Missing	895	1532	418	1103		
Potentially hazardous alcohol use (AUDIT-C) (*n* = 8093)						
Positive	1547 (55.2%)	2166 (60.4%)	301 (51.2%)	588 (53.8%)	0.640	<0.001
Negative	1258 (44.8%)	1418 (39.6%)	287 (48.8%)	505 (46.2%)		
Missing	1252	2100	661	1693		
STI symptoms currently (*n* = 10,430)						
Yes	332 (9.6%)	357 (7.6%)	222 (28.3%)	167 (11.6%)	<0.001	<0.001
No	3136 (90.4%)	4349 (92.4%)	562 (71.7%)	1278 (88.4%)		
Missing	589	978	465	1341		
STI (or partner with STI) in past month (*n* = 13,299)						
Yes	326 (8.1%)	303 (5.5%)	262 (22.5%)	219 (9.4%)	<0.001	<0.001
No	3424 (85.1%)	4878 (87.8%)	832 (71.5%)	1979 (84.5%)		
Unsure	273 (6.8%)	378 (6.8%)	69 (5.9%)	144 (6.2%)		
Missing	34	125	86	444		
PrEP-related Characteristics						
Prior PrEP use (*n* = 12,744)						
Yes	224 (18.2%)	385 (13.9%)	639 (16.2%)	1088 (19.7%)	0.108	<0.001
No	1008 (81.8%)	2377 (86.1%)	3299 (83.8%)	4443 (80.3%)		
Missing	119	153	17	24		
Disclosure of intended PrEP use (*n* = 8119)						
Yes	859 (37.8%)	1396 (44.0%)	418 (44.5%)	684 (40.0%)	<0.001	0.007
No	1411 (62.2%)	1778 (56.0%)	522 (55.5%)	1028 (60.1%)		
Missing	1787	2510	309	1074		
Sex Partner’s predicted reaction to PrEP (*n* = 13,517)						
Positive	2525 (64.0%)	3342 (60.4%)	598 (48.1%)	891 (32.3%)	<0.001	<0.001
Negative	172 (4.4%)	272 (4.9%)	117 (9.4%)	404 (14.6%)		
Unsure	1246 (31.6%)	1924 (34.7%)	528 (42.5%)	1468 (53.1%)		
Missing	114	146	6	23		

* Note percentages reflect non-missing observations. ** Percentages are column percentages calculated among non-missing observations. *p*-values are from Pearson χ^2^ tests (categorical) or t-tests (continuous) comparing mobile clinics versus fixed-site public health facilities within males and within females, respectively.

**Table 3:** Sociodemographic, behavioural, and clinical characteristics associated with PrEP initiation at a mobile clinic vs. public health facilities: univariate and multivariate analyses.

	Univariate Analysis		Multivariate Analysis	
	OR (95% CI)	*p*-Value	aOR (95% CI)	*p*-Value
Sociodemographic and Behavioural Covariates (all Participants, *n* = 13,807)				
Age [Ref: 15–19]				
Age 20–24	1.093 (0.994, 1.200)	0.065	1.293 (1.158, 1.445)	<0.001
Age 25–29	1.198 (1.083, 1.325)	<0.001	1.676 (1.476, 1.903)	<0.001
Age > 29	1.744 (1.543, 1.970)	<0.001	2.326 (1.933, 2.799)	<0.001
Gender [Ref: Male]				
Female	0.628 (0.586, 0.679)	<0.001	0.696 (0.622, 0.779)	<0.001
Gender minority	0.487 (0.236, 1.007)	0.052	0.662 (0.286, 1.533)	0.634
Relationship Status [Ref: Married]				
Single with partner (not married)	0.683 (0.569, 0.819)	<0.001	0.746 (0.604, 0.922)	0.007
Number of living children [Ref: None]				
≥1 child	0.771 (0.711, 0.836)	<0.001	0.600 (0.546, 0.661)	<0.001
Sex partner(s) living with HIV [Ref: No]				
Yes	0.400 (0.334, 0.481)	<0.001	0.276 (0.222, 0.342)	<0.001
Unsure	1.204 (1.111, 1.304)	<0.001	1.417 (1.295, 1.551)	<0.001
Partner ≥ 10 years older/younger than yourself [Ref: No]				
Yes	0.563 (0.480, 0.660)	<0.001	0.548 (0.458, 0.657)	<0.001
Unsure	0.391 (0.299, 0.513)	<0.001	0.352 (0.262, 0.472)	<0.001
Multiple partners in past month [Ref: No]				
Yes	0.933 (0.851, 1.024)	0.144		
Engaged in casual sex in past month [Ref: No]				
Yes	0.944 (0.859, 1.038)	0.234		
Partner has other sexual partners [Ref: No]				
Yes	0.770 (0.660, 0.899)	0.001	0.829 (0.692, 0.993)	0.041
Unsure	0.908 (0.834, 0.998)	0.025	0.791 (0.718, 0.871)	<0.001
Clinical covariates (females, *n* = 8470)				
STI symptoms currently [Ref: No]				
Yes	0.634 (0.523, 0.769)	<0.001	0.732 (0.552, 0.970)	0.030
STI/partner with STI in past month [Ref: No]				
Yes	0.575 (0.480, 0.688)	<0.001	0.634 (0.457, 0.880)	0.006
Unsure	1.073 (0.880, 1.307)	0.487	1.069 (0.768, 1.489)	0.692
Depressive Symptoms [Ref: None-Mild]				
Moderate- Severe	4.838 (3.496, 6.694)	<0.001	3.892 (2.437, 6.218)	<0.001
Potentially hazardous alcohol use [Ref: Neg]				
Positive	0.878 (1.311, 1.874)	0.128		
Pregnant [Ref: No/Not sure]				
Yes	0.052 (0.036, 0.073)	<0.001	0.089 (0.053, 0.152)	<0.001
Clinical covariates (males, *n* = 5306)				
STI symptoms currently [Ref: No]				
Yes	0.268 (0.221, 0.325)	<0.001	0.278 (0.215, 0.359)	<0.001
STI/partner with STI in past month [Ref: No]				
Yes	0.302 (0.253, 0.362)	<0.001	0.585 (0.432, 0.792)	<0.001
Unsure	0.961 (0.730, 1.265)	0.779	0.954 (0.625, 1.455)	0.827
Depressive Symptoms [Ref: None-Mild]				
Moderate–Severe	5.047 (3.347, 7.612)	<0.001	4.102 (2.561, 6.570)	<0.001
Potentially hazardous alcohol use [Ref: Neg]				
Positive	0.911 (0.737, 1.126)	0.391		
PrEP-related covariates (all participants, *n* = 13,465)				
Prior PrEP use [Ref: No]				
Yes	1.240 (1.121, 1.371)	<0.001	1.085 (0.977, 1.205)	0.126
Partner’s reaction to PrEP [Ref: Neg]				
Positive	4.510 (4.013, 5.295)	<0.001	4.528 (3.941, 5.204)	<0.001
Unsure	1.864 (1.623, 2.140)	<0.001	1.863 (1.621, 2.140)	<0.001

## Data Availability

Data are available from the corresponding author upon reasonable request.
